# Associations of selective serotonin reuptake inhibitors and long COVID risk in patients with depression: a retrospective cohort study

**DOI:** 10.1007/s15010-025-02648-z

**Published:** 2025-09-25

**Authors:** Zhenxiang Gao, Tomasz Tabernacki, Pamela B. Davis, David C. Kaelber, Rong Xu

**Affiliations:** 1 Center for Artificial Intelligence in Drug Discovery, School of Medicine, Case Western Reserve University, Cleveland, OH USA; 2 School of Medicine, Case Western Reserve University, Cleveland, OH USA; 3 Center for Community Health Integration, School of Medicine, Case Western Reserve University, Cleveland, OH USA; 4 Center for Clinical Informatics Research and Education, The Metro Health System, Cleveland, OH USA

**Keywords:** COVID-19, Long COVID, Selective serotonin reuptake inhibitors, Depression

## Abstract

**Purpose:**

To evaluate the potential of selective serotonin reuptake inhibitors (SSRIs) in reducing the risk of long COVID in patients with depression.

**Methods:**

This retrospective cohort study analyzed U.S. electronic health records from TriNetX platform to compare the risk of long COVID among adults with depression who were prescribed SSRIs versus non-SSRI antidepressants between March 2020 and December 2022. The main outcome was the long COVID diagnosis. As a sensitivity analysis, CDC-defined long COVID symptoms were used as alternative outcomes. Cox proportional hazards models were used to assess outcomes occurring 3–6 and 3–12 months after the index SARS-CoV-2 infection, with hazard ratios (HRs) and 95% confidence intervals (CIs) calculated.

**Results:**

After propensity score matching, the study included 31,264 patients, and the risk of long COVID diagnosis was significantly lower in the SSRI cohort compared to the matched non-SSRI antidepressant cohort, with hazard ratios of 0.57 (95% CI: 0.44–0.73) for the 3-6-month period and 0.59 (95% CI: 0.49–0.72) for the 3-12-month period. Sensitivity analyses in matched cohorts of 17,100 patients showed that SSRI use was associated with a significantly reduced risk of long COVID symptoms, consistent across symptom categories and pandemic periods.

**Conclusions:**

In adult patients with depression, SSRIs compared with non-SSRI antidepressants were associated with a lower risk of long COVID. These results offer preliminary evidence that SSRIs may help prevent long COVID in high‑risk populations and warrant further preclinical and clinical investigation.

**Supplementary Information:**

The online version contains supplementary material available at 10.1007/s15010-025-02648-z.

## Introduction

Long COVID, also known as post‑acute sequelae of SARS‑CoV‑2 infection (PASC), has rapidly evolved into one of the most pressing health crises of the post-pandemic era, affecting an estimated 65 million people worldwide and imposing enormous burdens on patients, healthcare systems, and societies [1, 2]. Defined by the World Health Organization (WHO) and Centers for Disease Control and Prevention (CDC) as persistent chronic symptoms emerging at least 3 months post-infection, long COVID commonly presents with debilitating fatigue, cognitive impairment (“brain fog”), dyspnea, and musculoskeletal pain [3, 4]. Despite its high prevalence, there is a lack of effective treatments for long COVID.

Depression appears to compound this challenge, possibly through shared neuroinflammatory pathways. SARS-CoV-2 infection triggers systemic and central inflammatory responses, correlating strongly with acute depressive symptoms [5–7]. Prospective cohorts show that patients who screen positive for depression one month after COVID-19 infection were more likely to report persistent physical and cognitive complaints at three months [8, 9]. Because depression itself is accompanied by immune dysregulation, timely treatment might lessen downstream inflammatory sequelae. Selective serotonin reuptake inhibitors (SSRIs), commonly prescribed as first-line treatments for depression, reduce neuroinflammation and have been observed to alleviate pro-inflammatory cytokine levels in clinical studies [10, 11]. We hypothesize that SSRI therapy at the time of infection had potential in preventing long‑COVID development.

Several studies have investigated the association between SSRI use and long COVID [12, 13]. A completed phase 2 trial of vortioxetine reported cognitive improvement in adults with post-acute COVID symptoms, especially in those with metabolic dysregulation and low-grade inflammation [14]. Meanwhile, additional SSRI treatment trials testing fluvoxamine and fluoxetine are ongoing [15]. Observational studies have shown inconsistent results. Analyses using the National COVID Cohort Collaborative (N3C) dataset indicated that SSRI use may reduce the risk of Long COVID [16, 17], whereas a study using the U.S. Veterans’ dataset found no such association [18]. These inconsistent findings may have resulted from different long COVID definitions, study periods, follow-up times, heterogeneous study populations, and non-active comparators.

In this study, we conducted a retrospective cohort study using electronic health records from the TriNetX database to investigate the association between SSRI use and the risk of long COVID compared with non-SSRI antidepressants in patients with depression. We also conducted extensive sensitivity analyses using two different definitions of long COVID, across different pandemic periods and follow-up durations. Additional subgroup analyses were performed based on symptom categories.

## Methods

### Data source

This study utilized data from TriNetX [19], a global federated health research network that provides continuously updated electronic health records (EHR) from over 100 million patients across 59 healthcare organizations in the United States. The patient distribution within the TriNetX network includes 25% from the Northeast, 17% from the Midwest, 41% from the South, and 12% from the West, with 5% of origins unknown. This retrospective study is exempt from informed consent. The data reviewed is a secondary analysis of existing data, does not involve intervention or interaction with human subjects, and is de-identified per the de-identification standard defined in Section § 164.514(a) of the HIPAA Privacy Rule. The process by which the data is de-identified is attested to through a formal determination by a qualified expert as defined in Section § 164.514(b)(1) of the HIPAA Privacy Rule. This formal determination by a qualified expert was refreshed in December 2020. Previously, we have utilized the TriNetX Analytics platform to conduct cohort studies, including evaluations of the potential clinical efficacy of drugs [20–25].

### Study population

The study cohort consisted of depression patients aged 18 years or older with documented COVID-19 infection, identified through either an encounter diagnosis code for COVID-19 or a positive SARS-CoV-2 antibody test result. COVID-19 diagnosis was identified using the ICD-10 code U07.1 or a positive RNA-based COVID-19 test recorded in TriNetX (code 9088, “SARS coronavirus 2 and related RNA”). Depression diagnoses were determined using ICD-10 codes for “Depressive Episode” (F32) and “Recurrent Major Depressive Disorder” (F33). The exposure was selective serotonin reuptake inhibitors (SSRIs), including citalopram, escitalopram, paroxetine, sertraline, fluvoxamine, fluoxetine, vortioxetine, and vilazodone. The comparison was with antidepressants other than SSRIs (non-SSRI antidepressants).

**Fig. 1:**
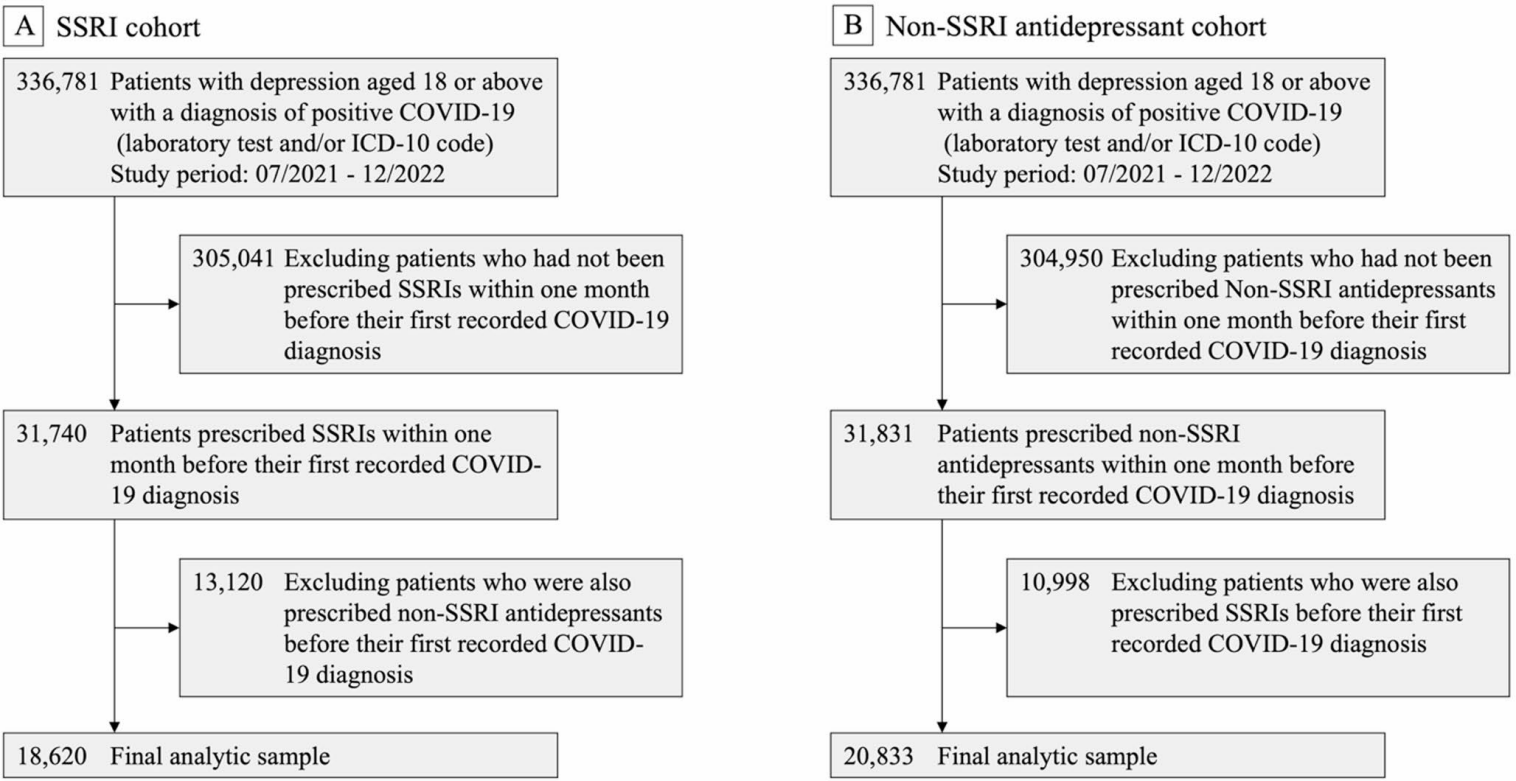
Flowchart of study cohort selection during the study period from July 2021 to December 2022

This study investigated the risk of long COVID in patients with preexisting depression following SARS-CoV-2 infection, focusing on the impact of SSRI use compared to non-SSRI antidepressants. The primary outcome, long COVID, was defined in TriNetX using the ICD diagnosis code U09.9 (“Post COVID-19 condition”). Since the ICD code U09.9 became effective on October 1, 2021, the study population included individuals who had a documented depression diagnosis with an active prescription for an antidepressant within one month prior to their first recorded COVID-19 diagnosis, between July 2021 and December 2022. The patients were categorized into two groups: (1) the SSRI group, consisting of individuals who were prescribed SSRIs without any other antidepressants, and (2) the non-SSRI antidepressant group, which included patients prescribed antidepressants but not SSRIs. Cohort selection details are illustrated in Fig. 1, which outlines the study design and inclusion criteria.

**Fig. 2:**
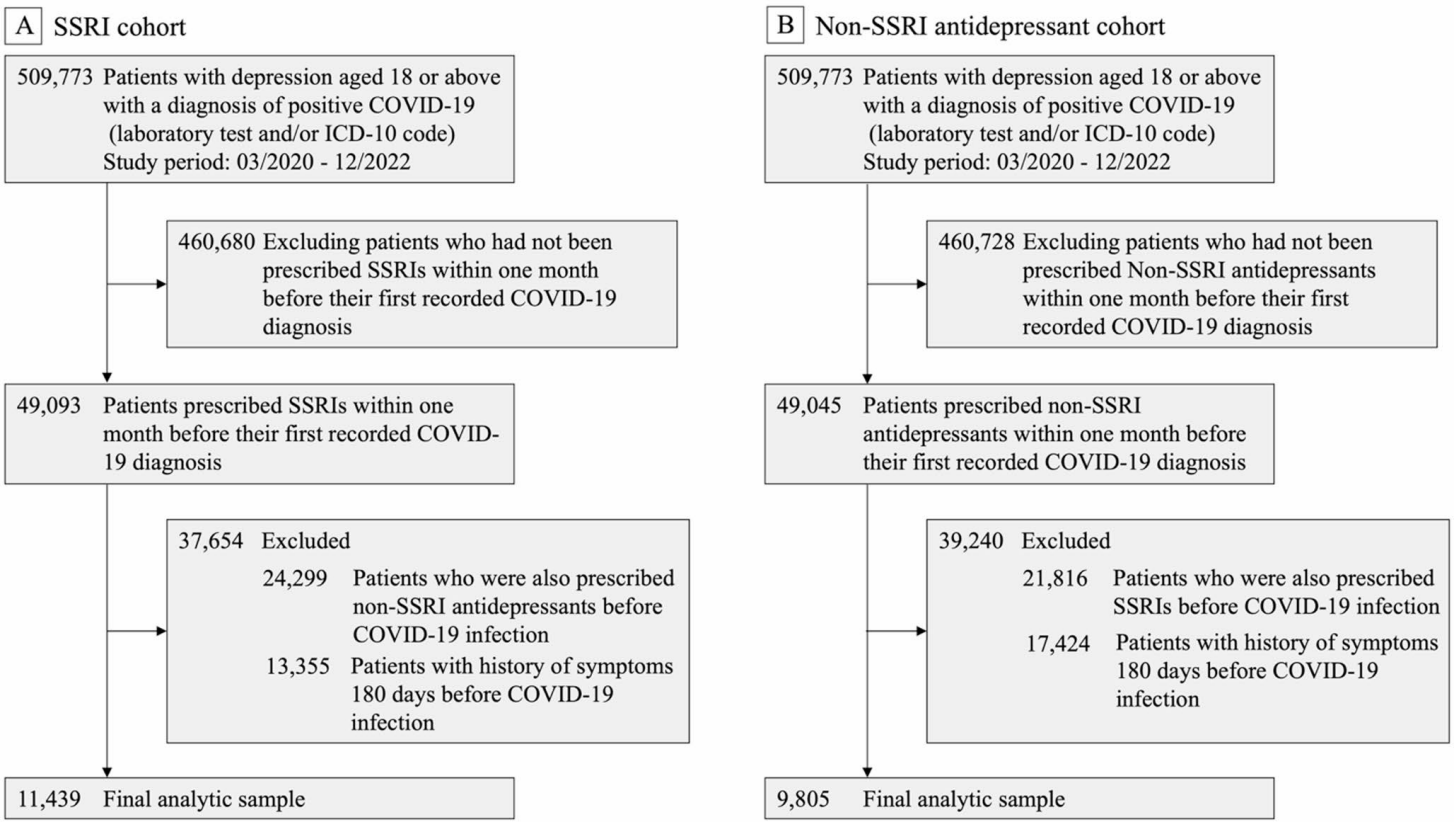
Flowchart of study cohort selection during the study period from March 2020 to December 2022

For sensitivity analyses, we used long COVID symptoms defined by the CDC as a range of symptoms or conditions that occur after SARS-CoV-2 infection and persist for at least three months [24]. Patient data were collected from March 2020 through December 2022. The SSRI group consisted of individuals with a documented diagnosis of depression who were prescribed SSRIs within one month prior to their first recorded COVID-19 diagnosis. The non-SSRI antidepressant group comprised patients prescribed other antidepressants during the same pre-infection window. Both groups had no history of long COVID symptoms within 180 days before SARS-CoV-2 infection. Figure 2 illustrates the details of cohort selection. For subgroup analyses, the study period was stratified into two phases based on the dominant SARS-CoV-2 variants: March 2020 to June 2021, primarily characterized by Alpha and Beta variants, and July 2021 to December 2022, predominantly marked by Delta and Omicron variants. Additionally, we categorized long COVID symptoms into groups, including general symptoms, respiratory and cardiovascular symptoms, neurological symptoms, digestive symptoms, and other symptoms including skin rashes, joint or muscle pain. We separately examined the associations of SSRIs with each of these symptom groups. Details of specific ICD-10 codes for identifying these groups are provided in Supplementary Table S1 [26]. Since this study specifically focused on patients with depression, depression and anxiety symptoms were separated from the neurological category and analyzed as a distinct group. We further evaluated consistency across different drug exposure windows and compared outcomes between SSRIs with and without sigma-1 receptor (S1R) agonist activity, because previous studies suggest that S1R agonism may be a mechanism by which SSRIs reduce inflammatory responses [17].

Propensity score matching was applied to balance key characteristics between the exposure and control groups. Covariates were selected based on their relevance to long COVID risk [16, 17, 27–29] and included demographics (age, sex, and ethnicity), socioeconomic factors (education level, employment status, social and psychosocial environment, and housing conditions), problems related to lifestyle, and psychiatric comorbidities (schizophrenia, mood disorders, and anxiety disorders). Additionally, we controlled for chronic health conditions such as hypertension, diabetes, heart disease, cerebrovascular disease, respiratory diseases, cancer, kidney disease, liver disease, nicotine dependence, alcohol addiction, and obesity. We also adjusted for prior hospital inpatient and observation care services, medication history, including prescriptions associated with COVID-19. The list of covariates is included in Supplementary Table S2.

### Statistical analyses

To evaluate differences in long COVID risk between patients prescribed SSRIs and those prescribed non-SSRI antidepressants, hazard ratios (HRs) with 95% confidence intervals (CIs) were estimated using Cox proportional hazards regression models to quantify the relative risk between the two treatment groups. Cumulative incidences were estimated using Kaplan-Meier survival analysis. The index event, marking the start of follow-up, was defined as the first recorded COVID-19 diagnosis. Patients in the matched cohorts were followed until the occurrence of the outcome, death, loss to follow-up, or the end of the predefined follow-up windows (3–6 months and 3–12 months post-index), whichever occurred first. All statistical analyses were conducted using the TriNetX Analytics platform. Statistical significance was determined using a two-sided *p*-value threshold of < 0.05, with analyses finalized in April 2025.

## Results

**Fig. 3:**
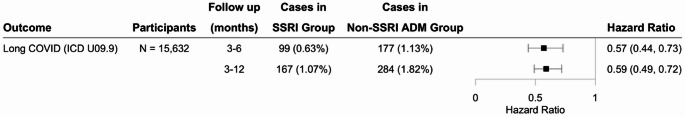
Comparison of long COVID (ICD code U09.9) risk between propensity-score matched groups of patients with depression prescribed SSRIs versus non-SSRI antidepressants following initial SARS-CoV-2 infection between July 2021 and December 2022

**Fig. 4:**
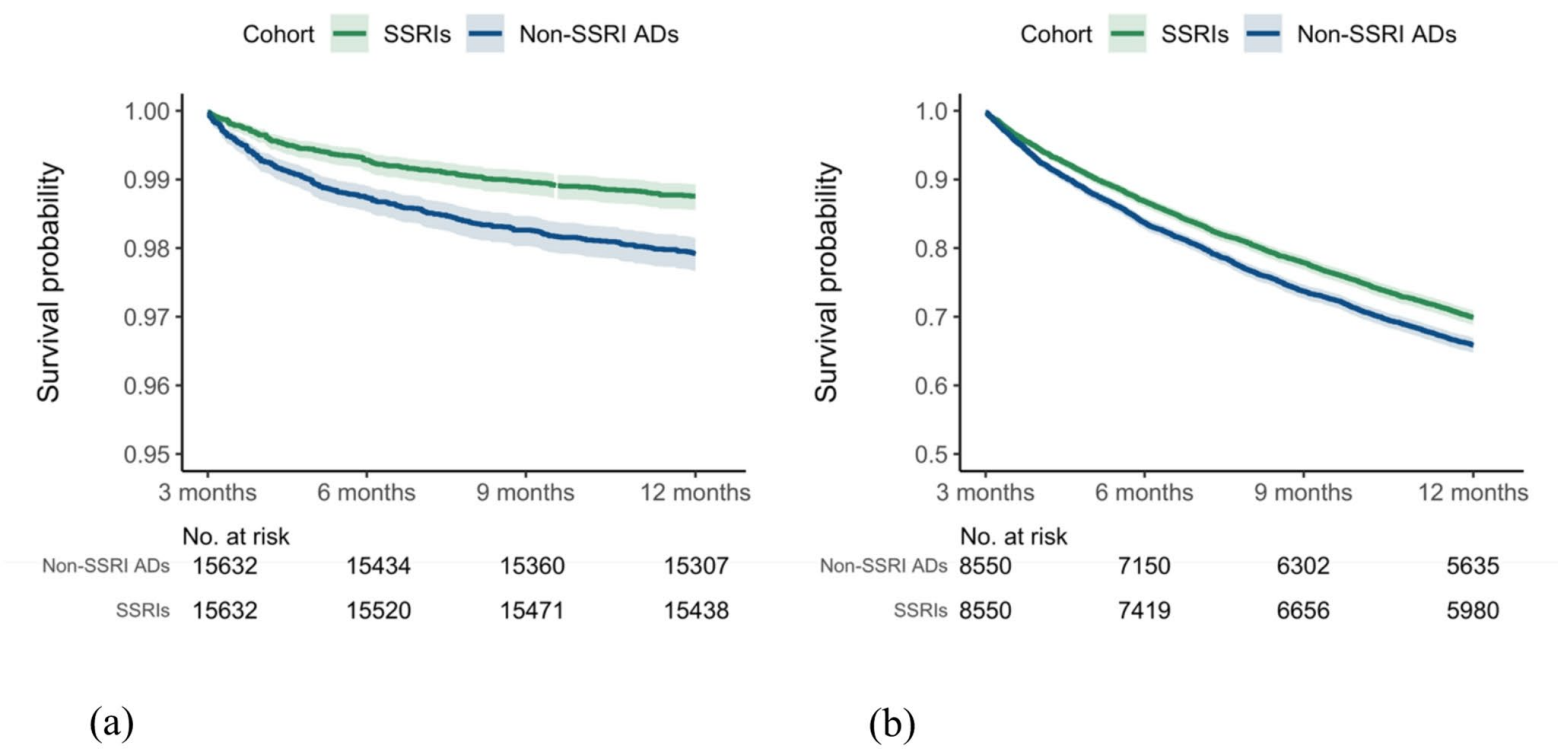
Kaplan-Meier plots of long COVID-free survival among patients prescribed SSRIs versus non-SSRI antidepressants: (**a**) ICD U09.9 diagnosis (July 2021-December 2022); (**b**) long COVID symptoms (March 2020-December 2022)

Using the ICD-10 diagnosis code-based definition of long COVID for the study period from July 2021 to December 2022, the initial population comprised 18,620 patients prescribed SSRIs, and 20,833 patients prescribed non-SSRI antidepressants between July 2021 and December 2022 (Table 1). Compared to the SSRI group, those in the non-SSRI cohort had a higher proportion of males and African Americans and a higher prevalence of heart failure, hypertension, respiratory diseases, alcohol/nicotine addiction, and obesity. After propensity-score matching, the two cohorts were balanced. As illustrated in Fig. 3, patients receiving SSRIs exhibited a significantly lower hazard ratio for developing long COVID after COVID-19 infection compared to those prescribed non-SSRI antidepressants, with hazard ratios of 0.57 (95% CI: 0.44–0.73) and 0.59 (95% CI: 0.49–0.72) at follow-up periods of 3 to 6 months and 3 to 12 months, respectively. The Kaplan-Meier curve for the study cohort with the outcome of long COVID using the ICD-10 diagnosis code was shown in Fig. 4(a). Consistent protective associations were observed in analyses using alternative drug exposure windows (Figure S1 in Supplementary File). Furthermore, no significant reduction in long COVID risk was observed between patients prescribed S1R agonist SSRIs and those prescribed other SSRIs without S1R agonist activity (Figure S2 in Supplementary File).

**Table 1:** The characteristics of patients with depression who were prescribed SSRIs or non-SSRI antidepressants during the study period (September 2021 to December 2022), both before and after propensity score matching for covariates

Characteristics	Before Matching			After Matching		
	Exposure Cohort	Control Cohort	SMD	Exposure Cohort	Control Cohort	SMD
Total No.	18,620	20,833		15,632	15,632	
Age	52.9(21.4)	56.9(17.3)	0.20*	56.3(20.4)	55.8(17.8)	0.02
***Sex***, %						
Female	68.6	67.1	0.03	67.5	67.5	0.001
Male	28.0	29.9	0.04	29.4	29.3	0.0005
***Ethnicity***, %						
Hispanic/Latinx	6.5	5.9	0.02	6.1	5.9	0.008
Not Hispanic/Latinx	75.1	78.2	0.07	76.5	76.7	0.006
***Race***, %						
African American/Black	8.7	10.8	0.07	9.5	9.4	0.001
White	77.3	77.3	0.001	77.8	77.6	0.004
Asian	1.8	1.3	0.04	1.5	1.5	0.005
***Medical History***, %						
Pneumonia, unspecified organism	15.3	19.4	0.11*	16.7	16.9	0.005
Chronic lower respiratory diseases	35.7	43.6	0.16*	38.9	38.8	0.002
Chronic obstructive pulmonary disease	13.3	18.8	0.15*	15.4	15.8	0.009
Asthma	21.1	25.7	0.11*	22.6	22.8	0.004
Mental disorders due to known physiological conditions	10.5	13.6	0.09	11.9	12.0	0.003
Anxiety	69.1	70.9	0.04	68.4	69.1	0.014
Alcohol related disorders	6.6	11.0	0.15*	7.6	7.9	0.01
Nicotine dependence	17.7	27.3	0.23*	20.7	21.3	0.01
Hypertensive diseases	50.2	62.4	0.24*	57.2	56.6	0.01
Diabetes mellitus	24.5	31.4	0.15*	27.9	27.7	0.005
Cerebral infarction	6.7	8.0	0.05	7.5	7.5	0.001
Acute myocardial infarction	6.1	8.0	0.07	6.9	7.1	0.005
Pulmonary embolism	3.5	5.5	0.09	4.1	4.2	0.007
Heart failure	14.1	17.9	0.11*	16.0	16.1	0.001
Acute kidney failure and chronic kidney disease	20.5	27.3	0.16*	23.5	23.6	0.003
Diseases of liver	12.9	20.0	0.19*	15.0	15.4	0.009
Malignant neoplasm	1.7	2.5	0.05	1.9	2.0	0.005
Gastro-esophageal reflux disease	39.2	50.7	0.23*	44.3	44.6	0.005
Overweight and obesity	35.2	43.3	0.16*	39.2	38.9	0.006
Hypothyroidism	18.7	23.9	0.12*	21.1	21.3	0.005
Osteoporosis	10.0	12.9	0.09	11.4	11.4	0.001
Rheumatoid arthritis	2.9	5.7	0.13*	3.5	3.6	0.004
SARS-CoV-2 (COVID-19) Vaccine, %	22.6	24.3	0.03	23.1	23.4	0.006
Adverse socioeconomical determinants of health, %	8.4	11.3	0.10*	9.1	9.1	0.001
Problems related to lifestyle, %	8.9	14.1	0.16*	10.3	10.5	0.008
Hospital inpatient and observation care Services, %	29.0	38.4	0.19*	32.8	32.9	0.003
***Drug use***, %						
Antipsychotics	19.6	33.4	0.31*	23.2	23.8	0.012
Ritonavir	1.2	1.6	0.03	1.4	1.4	0.0005
Nirmatrelvir	1.2	1.4	0.02	1.3	1.3	0.002
Remdesivir	0.17	0.16	0.002	0.19	0.17	0.006
Baricitinib	0.05	0.05	0.002	0.06	0.06	0
Tocilizumab	0.18	0.33	0.02	0.21	0.24	0.006

Note: SMD - standardized mean difference. *SMD > 0.1, a threshold for declaring imbalance

**Fig. 5:**
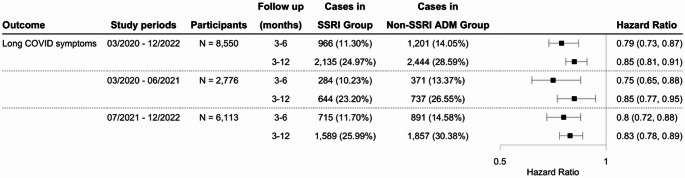
Comparison of the risk of long COVID symptoms between propensity-score matched groups of patients with depression prescribed SSRIs versus other antidepressants following initial SARS-CoV-2 infection

**Fig. 6:**
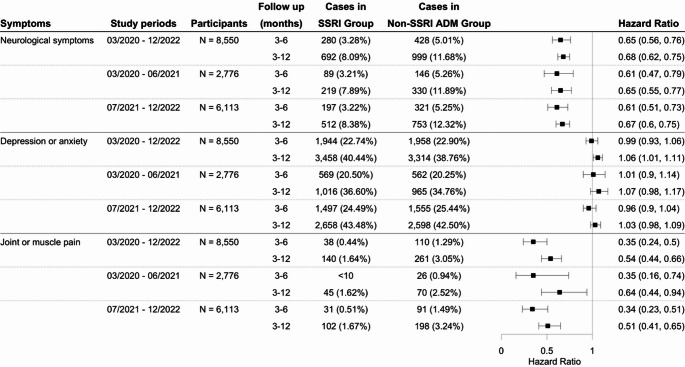
Comparison of the risk of neurological symptoms, joint/muscle pain, and depression/anxiety symptoms between propensity-score matched groups of patients with depression prescribed SSRIs versus other antidepressants following initial SARS-CoV-2 infection

Using the long COVID definition based on the CDC definition for the study period from March 2020 and December 2022, this study identified an initial cohort of 11,439 patients who received SSRIs, and 9,850 patients prescribed non-SSRI antidepressants. The characteristics of the matched cohorts are presented in Supplementary Table S3. SSRI was significantly associated with a reduced risk of long COVID-related symptoms compared to non-SSRI antidepressants, with hazard ratios of 0.79 (95% CI: 0.73–0.87) and 0.85 (95% CI: 0.81–0.89) at 3–6 and 3–12 months of follow-up, respectively (Fig. 5). Figure 4(b) presents the Kaplan-Meier curve for the study cohort with long COVID symptoms as the outcome. This reduced risk remained consistent when the analysis was stratified by study period according to the dominant SARS-CoV-2 variants (Fig. 5). Sensitivity analyses by the symptom group revealed that patients prescribed SSRIs had a lower risk of developing neurological symptoms and joint or muscle pain compared to those receiving non-SSRI antidepressants (Fig. 6), but not depression or anxiety symptoms. Additional details of other symptoms are provided in Figure S3 of the supplementary file. Analyses using different drug exposure windows showed consistent protective associations, while no significant difference in long COVID risk was observed between patients prescribed S1R agonist SSRIs versus SSRIs without S1R agonist activity (Figures S4-S5, Supplementary File).

## Discussion

In this study, we conducted a retrospective observational study that showed that patients with depression who were prescribed SSRIs exhibited a significantly lower risk of long COVID - particularly neurological and musculoskeletal symptoms - after SARS-CoV-2 infection compared to matched patients prescribed non-SSRI antidepressants. These findings were consistent across different long COVID definitions, follow-up intervals, and SARS-CoV-2 variant periods.

Our study findings align with recent large-scale observational evidence suggesting SSRIs may be associated with a reduced risk of developing long COVID after SARS-CoV-2 infection. A retrospective study of 302,626 patients with a diagnosis of depression before COVID diagnosis found that SSRI use was associated with a risk ratio of 0.92 (95% CI: 0.86, 0.99) for diagnosis with long COVID compared to those not using an SSRI [16]. Similarly, in a study of 17,908 patients with COVID-19 from the National COVID Cohort Collaborative (N3C), individuals exposed to S1R agonist SSRIs showed a 29% reduction in relative risk of long-COVID [17]. In contrast, one study analyzing the U.S. veterans’ dataset reported no significant association between SSRI use and the risk of long COVID [18]. However, this study included a heterogeneous cohort of patients with anxiety, depression, or post-traumatic stress disorder (PTSD) and compared users of SSRIs with patients who did not use any antidepressants, which could suffer confounding effects by indications. Our study included 31,264 COVID patients with depression and compared patients prescribed SSRIs to those receiving non-SSRI antidepressants. We found that SSRI use was associated with a reduced risk of long COVID compared to non-SSRI antidepressants. Taken together, findings from our study suggest that SSRIs may have a protective effect in preventing or delaying the development of long COVID in patients with depression following COVID-19 infection.

In an exploratory cohort of 95 post-COVID-19 patients treated with SSRIs, Rus et al. observed substantial symptom amelioration across neuropsychiatric and musculoskeletal domains: “brain fog” and sensory overload scores decreased by an average of 3.8 and 3.6 points respectively, and Bell functional scores nearly doubled from 23.5 to 47.2 within 4–6 weeks of SSRI initiation [12]. These findings mirror our subgroup analyses, which showed that SSRI initiation prior to SARS-CoV-2 infection was chiefly associated with reduced neurological manifestations (e.g., cognitive impairment, headache, sleep disturbance) and musculoskeletal pain, while affective, constitutional, gastrointestinal, and dermatological PASC symptoms were unchanged. By contrast, our propensity-matched comparison of SSRI versus non-SSRI antidepressant users revealed no significant difference in rates of ongoing depression or anxiety symptoms. This diverges from earlier work by Mazza et al., who reported a 92% response (≥ 50% reduction on Hamilton Depression Rating Scale) in 60 post-COVID depression patients treated with SSRIs versus non-users [13]. However, this study only compared SSRIs against SSRI-naïve groups, whereas we compared versus individuals receiving other antidepressant medications.

SSRIs modulate both innate and adaptive immune pathways in ways that could attenuate the persistent inflammation underlying long-COVID. Long COVID is characterized by persistent immune activation with elevated proinflammatory cytokines, chemokines, and type I interferon signatures that correlate with prolonged neurocognitive and somatic symptoms [30, 31]. SSRIs possess well-described immunomodulatory properties: they suppress proinflammatory cytokine release, restore IFN/IL-10 balance, deplete platelet serotonin to limit neutrophil endothelial adhesion, and modulate lymphocyte signaling [32, 33]. Clinical meta-analyses further show SSRI therapy reduces circulating IL-6, IL-1β, and TNF-α [34, 35]. Though further work is needed to further characterize long-COVID associated immunomodulatory mechanisms, there is a plausible biological rationale for SSRIs to attenuate the maladaptive inflammation of long COVID and thereby decrease its incidence and severity.

### Strengths and limitations

Our study has several key strengths. First, we employed both a clinician-coded U09.9 diagnosis and the CDC symptom-based definition of long COVID to ensure robustness of our overall findings. Second, our findings were consistent across multiple SARS-CoV-2 variant periods and follow-up intervals.

Several limitations of our analysis merit consideration. First, the retrospective EHR-based observational study has limitations of residual confounding and biases, therefore causality cannot be established. Second, our primary outcome (clinician-coded long COVID via ICD-10 U09.9) became available only in October 2021 and underestimated the true incidence of long COVID. For sensitivity analysis, we used the CDC symptom-based definition, and the overall findings were consistent for these two long COVID definitions. Third, details on SSRI dose, duration, and adherence were not able to be ascertained from patient EHRs. In addition, our cohort is restricted to patients with diagnosed depression engaged in healthcare systems contributing to TriNetX. The generalizability of our findings needs to be validated in other populations or other databases.

## Conclusions

In summary, our findings suggest that SSRIs were associated with a lower risk of developing long-COVID, particularly its neurologic and musculoskeletal sequelae, in patients with depression, compared with non-SSRI antidepressants. If confirmed in prospective trials, this could identify SSRIs as readily available, well-tolerated agents for long-COVID mitigation. Future work should include randomized clinical trials to establish causality and mechanistic investigations.

## Supplementary Information

Below is the link to the electronic supplementary material.

Supplementary Material 1

## Data Availability

All data relevant to the study were using the TriNetX platform (https://trinetx.com/).
