# Supplementary material for: Associations of selective serotonin reuptake inhibitors and long COVID risk in patients with depression: a retrospective cohort study
Source: Infection. 2025 Sep 25;54(1):203–11. doi: 10.1007/s15010-025-02648-z (PMC12771409; doi:10.1007/s15010-025-02648-z)

Supporting Information

**Table S1**. Names and codes of symptoms commonly linked to long COVID

| **Symptom group** | **Name** | **code** |
| --- | --- | --- |
| General symptoms | Tiredness or fatigue that interferes with daily life | R53.83 |
|  | Fever | R50.9 |
| Respiratory and heart symptoms | Shortness of breath | R06.02 |
|  | Coughing | R05 |
|  | Chest pain | R07.9 |
|  | Fast-beating or pounding heart (heart palpitations) | R00.2 |
| Neurological symptoms | Symptoms and signs involving cognitive functions and awareness | R41.8 |
|  | Headaches | R51 |
|  | Sleep disturbances (insomnia) | G47.00 |
|  | Dizziness when you stand up (lightheadedness) | R42 |
|  | Change in smell or taste | R43.8 |
|  | Pins-and-needles feelings (paresthesia) | R20.2 |
| Digestive Symptoms | Diarrhea | R19.7 |
|  | Stomach pain (abdominal pain) | R10.9 |
|  | Constipation | K59.00 |
| Rashes | Skin rashes | R21 |
| Joint or muscle pain | Joint pain (arthralgia) | M25.50 |
|  | Muscle aches (myalgia) | M79.1 |

**Table S2**. Covariates and their standardized names and codes

| **Covariate** | **Description** | **Abbreviation or code** |
| --- | --- | --- |
| Age | Age at index | AI |
| Sex | Female | F |
|  | Male | M |
| Ethnicity | Hispanic/Latinx | 2135-2 |
|  | Not Hispanic/Latinx | 2186-5 |
| Race | African American/Black | 2054-5 |
|  | White | 2106-3 |
|  | Asian | 2028-9 |
| SARS-CoV-2 (COVID-19) Vaccine | SARS-CoV-2 (COVID-19) Vaccine | 213 |
| Socioeconomic status | Persons with potential health hazards related to socioeconomic and psychosocial circumstances | Z55-Z65 |
| Problems related to lifestyle | Problems related to lifestyle | Z72 |
| Hospitalization | Hospital Inpatient and Observation Care Services | 1013659 |
| Pre-existing medical conditions | Pneumonia, unspecified organism | J18 |
|  | Chronic lower respiratory diseases | J40-J4A |
|  | Other chronic obstructive pulmonary disease | J44 |
|  | Asthma | J45 |
|  | Mental disorders due to known physiological conditions | F01-F09 |
|  | Depressive episode | F32 |
|  | Major depressive disorder, recurrent | F33 |
|  | Anxiety, dissociative, stress-related, somatoform and other nonpsychotic mental disorders | F40-F48 |
|  | Alcohol related disorders | F10 |
|  | Nicotine dependence | F17 |
|  | Hypertensive diseases | I10-I1A |
|  | Diabetes mellitus | E08-E13 |
|  | Cerebral infarction | I63 |
|  | Acute myocardial infarction | I21 |
|  | Pulmonary embolism | I26 |
|  | Heart failure | I50 |
|  | Acute kidney failure and chronic kidney disease | N17-N19 |
|  | Diseases of liver | K70-K77 |
|  | Malignant neoplasm without specification of site | C80 |
|  | Gastro-esophageal reflux disease | K21 |
|  | Overweight and obesity | E66 |
|  | Other hypothyroidism | E03 |
|  | Osteoporosis without current pathological fracture | M81 |
|  | Rheumatoid arthritis, unspecified | M06.9 |
| Pre-existing anti-diabetes medicine | Insulins | A10A |
|  | Biguanides | A10BA |
|  | Sulfonylureas | A10BB |
|  | Alpha glucosidase inhibitors | A10BF |
|  | Thiazolidinediones | A10BG |
|  | Dipeptidyl peptidase 4 (DPP-4) inhibitors | A10BH |
|  | Other blood glucose lowering drugs, excl. insulins | A10BX |
| Pre-existing medicine | Antipsychotics | CN700 |
|  | Ritonavir | 85762 |
|  | Nirmatrelvir | 2587892 |
|  | Remdesivir | 2284718 |
|  | Baricitinib | 2047232 |
|  | Tocilizumab | 612865 |

**Table S3**. The characteristics of patients with depression who were prescribed SSRIs or other antidepressants and had no history of long COVID symptoms within 180 days prior to SARS-CoV-2 infection during the study period (March 2020 to December 2022), both before and after propensity score matching for covariates.

| **Characteristics** | **Before Matching** | | | **After Matching** | | |
| --- | --- | --- | --- | --- | --- | --- |
|  | **Exposure Cohort** | **Control Cohort** | **SMD** | **Exposure Cohort** | **Control Cohort** | **SMD** |
| Total No. | 11,439 | 9,805 |  | 8,550 | 8,550 |  |
| Age | 50.1(21.3) | 55.3 (17.5) | 0.26* | 55.4(19.9) | 54.4(17.7) | 0.05 |
| ***Sex,*** % | | | | | | |
| Female | 68.7 | 66.5 | 0.04 | 66.9 | 67.3 | 0.008 |
| Male | 28.7 | 31.1 | 0.05 | 30.6 | 30.3 | 0.006 |
| ***Ethnicity,*** % | | | | | | |
| Hispanic/Latinx | 6.8 | 5.9 | 0.03 | 5.9 | 6.1 | 0.008 |
| Not Hispanic/Latinx | 69.9 | 71.0 | 0.02 | 70.4 | 70.3 | 0.003 |
| ***Race,*** % | | | | | | |
| African American/Black | 8.4 | 9.2 | 0.02 | 8.8 | 8.8 | 0 |
| White | 77.6 | 77.8 | 0.005 | 78.0 | 77.9 | 0.002 |
| Asian | 1.8 | 1.3 | 0.04 | 1.4 | 1.4 | 0.001 |
| ***Medical History,*** % | | | | | | |
| Pneumonia, unspecified organism | 9.5 | 10.4 | 0.03 | 9.8 | 10.1 | 0.009 |
| Chronic lower respiratory diseases | 28.4 | 32.4 | 0.08 | 29.9 | 30.5 | 0.01 |
| Chronic obstructive pulmonary disease | 8.5 | 11.9 | 0.11* | 10.3 | 10.3 | 0.0007 |
| Asthma | 17.6 | 19.6 | 0.05 | 18.1 | 18.6 | 0.01 |
| Mental disorders due to known  physiological conditions | 7.5 | 9.2 | 0.06 | 8.8 | 8.4 | 0.01 |
| Anxiety | 63.0 | 61.7 | 0.02 | 59.9 | 60.6 | 0.01 |
| Alcohol related disorders | 5.2 | 8.8 | 0.14* | 6.6 | 6.8 | 0.007 |
| Nicotine dependence | 13.3 | 20.5 | 0.19* | 16.3 | 17.1 | 0.02 |
| Hypertensive diseases | 40.7 | 51.6 | 0.22* | 49.5 | 48.6 | 0.01 |
| Diabetes mellitus | 18.5 | 23.4 | 0.12* | 22.3 | 22.2 | 0.003 |
| Cerebral infarction | 4.0 | 4.6 | 0.02 | 4.7 | 4.6 | 0.004 |
| Acute myocardial infarction | 3.1 | 3.8 | 0.04 | 3.7 | 3.6 | 0.008 |
| Pulmonary embolism | 2.0 | 2.3 | 0.02 | 2.4 | 2.3 | 0.008 |
| Heart failure | 8.2 | 9.5 | 0.04 | 9.8 | 9.2 | 0.01 |
| Acute kidney failure and chronic kidney  disease | 13.5 | 17.4 | 0.11* | 16.0 | 16.2 | 0.005 |
| Diseases of liver | 7.9 | 12.3 | 0.14* | 10.0 | 10.4 | 0.01 |
| Malignant neoplasm | 0.93 | 1.1 | 0.02 | 1.1 | 1.1 | 0.001 |
| Gastro-esophageal reflux disease | 29.6 | 36.9 | 0.15* | 34.2 | 34.4 | 0.004 |
| Overweight and obesity | 28.3 | 34.3 | 0.12* | 32.7 | 32.4 | 0.004 |
| Hypothyroidism | 15.5 | 19.4 | 0.11* | 18.2 | 18.0 | 0.006 |
| Osteoporosis | 6.9 | 8.1 | 0.04 | 8.1 | 7.9 | 0.005 |
| Rheumatoid arthritis | 2.3 | 3.5 | 0.07 | 2.9 | 3.0 | 0.006 |
| SARS-CoV-2 (COVID-19) Vaccine, % | 15.9 | 16.7 | 0.02 | 16.1 | 16.2 | 0.003 |
| Adverse socioeconomical determinants of  health, % | 5.9 | 6.6 | 0.02 | 5.8 | 6.1 | 0.01 |
| Problems related to lifestyle, % | 5.5 | 8.5 | 0.11* | 6.6 | 6.9 | 0.01 |
| Hospital inpatient and observation care  Services, % | 17.5 | 21.8 | 0.11* | 19.6 | 19.9 | 0.007 |
| ***Drug use,*** % |  |  |  |  |  |  |
| Antipsychotics | 14.6 | 26.7 | 0.30* | 19.1 | 20.3 | 0.03 |
| Ritonavir | 0.90 | 1.33 | 0.04 | 1.1 | 1.1 | 0.004 |
| Nirmatrelvir | 0.8 | 1.16 | 0.03 | 1.01 | 0.99 | 0.001 |
| Remdesivir | 0.17 | 0.20 | 0.008 | 0.21 | 0.20 | 0.002 |
| Baricitinib | 0.09 | 0.10 | 0.004 | 0.12 | 0.12 | 0 |
| Tocilizumab | 0.09 | 0.10 | 0.004 | 0.12 | 0.12 | 0 |

Note: SMD - standardized mean difference. *SMD > 0.1, a threshold for declaring imbalance.

**Figure S1**. Sensitivity analysis of long COVID (ICD code U09.9) risk among patients with depression, comparing SSRI versus non-SSRI antidepressant use under alternative drug exposure windows between July 2021 and December 2022.


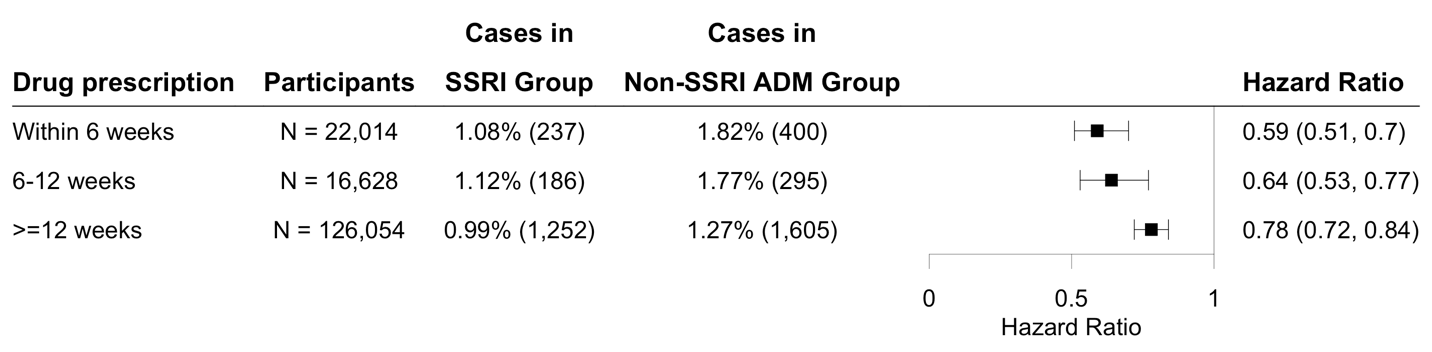


**Figure S2**. Comparison of long COVID (ICD code U09.9) risk between propensity-score matched groups of patients with depression prescribed S1R agonist SSRI versus SSRI without S1R agonist activity following initial SARS-CoV-2 infection between July 2021 and December 2022.


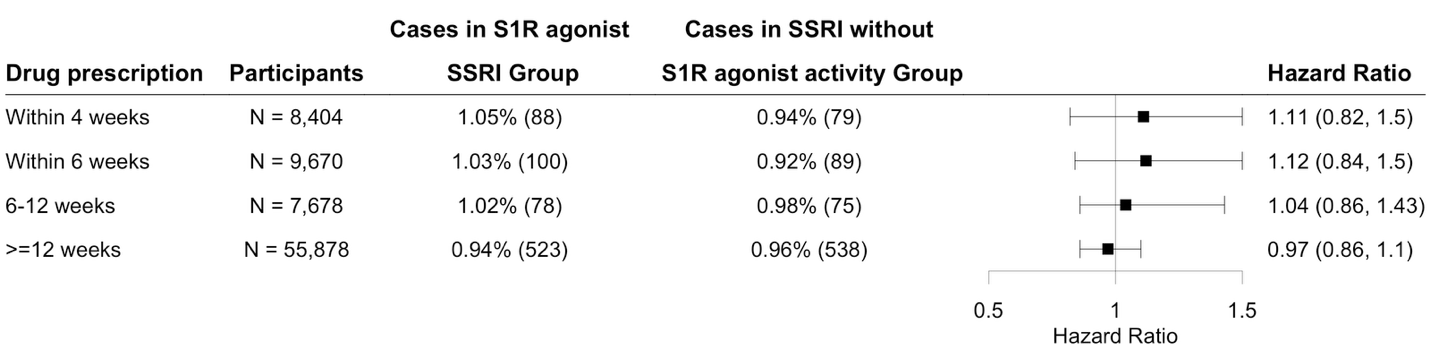


**Figure S3**. Comparison of other long COVID symptoms between propensity-score matched groups of patients with depression prescribed SSRIs versus other antidepressants following initial COVID-19 infection.


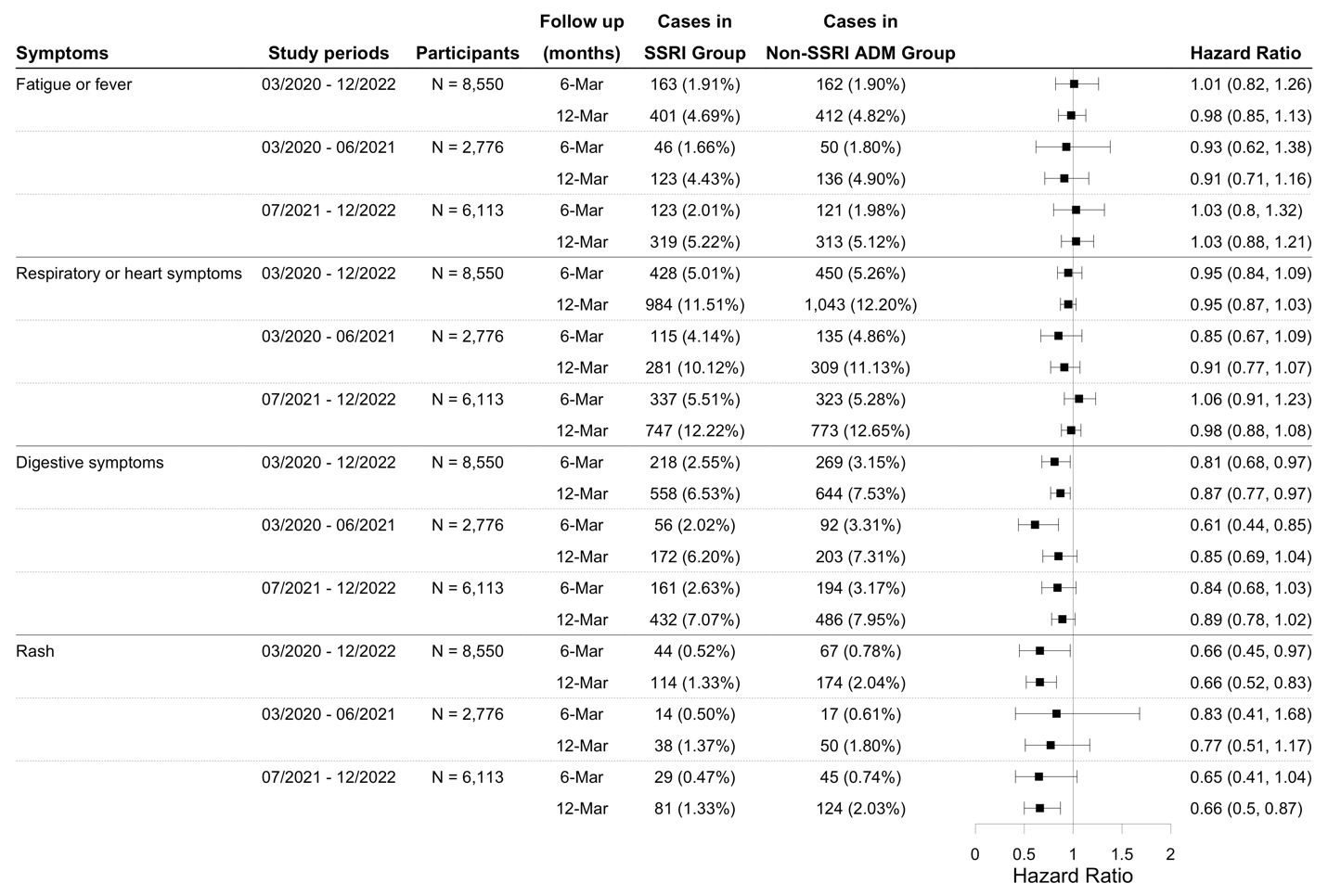


**Figure S4**. Sensitivity analysis of long COVID symptoms risk among patients with depression, comparing SSRI versus non-SSRI antidepressant use under alternative drug exposure windows between March 2020 and December 2022.


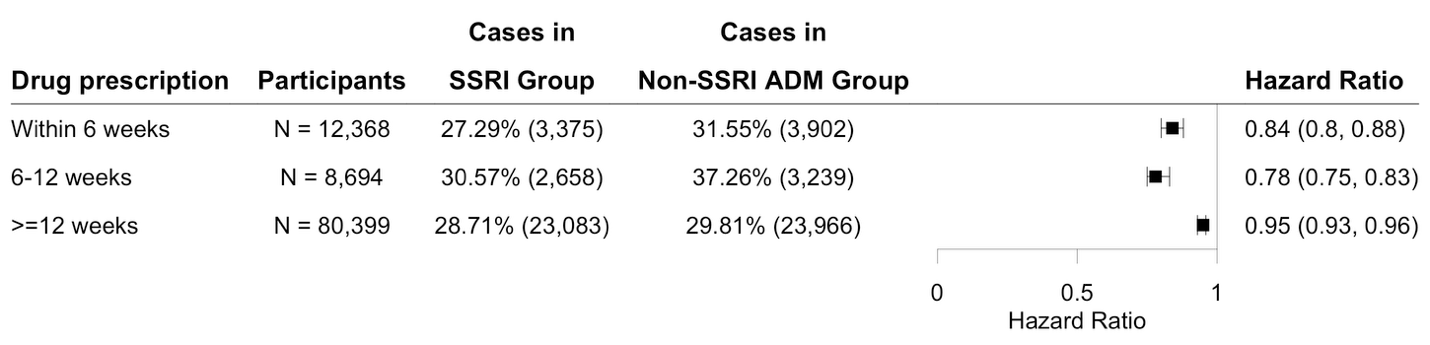


**Figure S5**. Comparison of long COVID symptoms risk between propensity-score matched groups of patients with depression prescribed S1R agonist SSRI versus SSRI without S1R agonist activity following initial SARS-CoV-2 infection between March 2020 and December 2022.


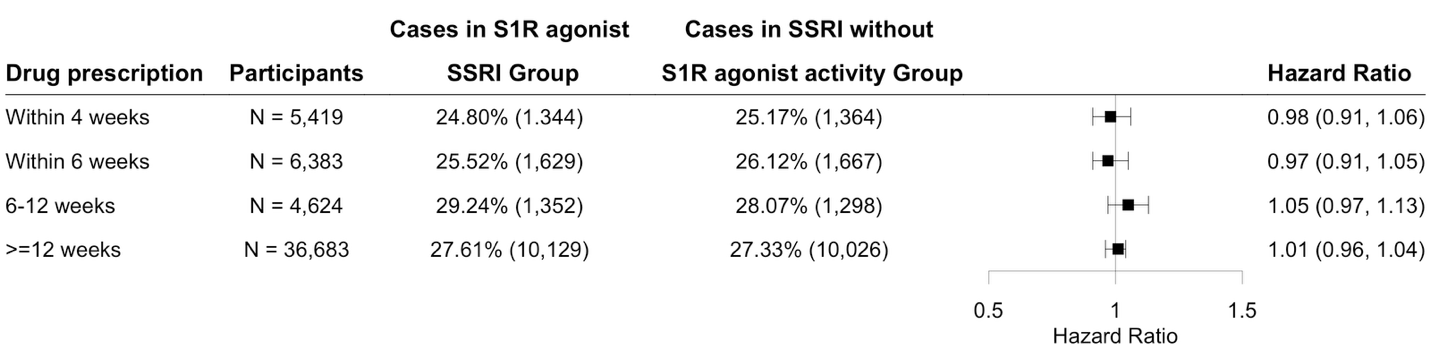

Supplement: Supplementary file 1 — Supplementary Material 1 [file 15010_2025_2648_MOESM1_ESM.docx]
